# Pneumococcal carriage prevalence, serotype distribution, and vaccine coverage in Ethiopia 12 years after pneumococcal vaccine introduction

**DOI:** 10.1016/j.vaccine.2025.127762

**Published:** 2025-09-17

**Authors:** Hiwot Yigzaw Bizuayehu, Yohannes Kebede, Merga Deressa, Esrael Solomon, Dadi Marami, Angela Karani, Elizabeth Gardiner, Yadeta Dessie, J. Anthony G. Scott, Katherine E. Gallagher

**Affiliations:** a Hararghe Health Research Partnership, Haramaya University, Harar, Ethiopia; b Department of Infectious Disease Epidemiology, London School of Hygiene & Tropical Medicine, London, UK; c Faculty of Public Health, Jimma University; d College of Health and Medical Sciences, Haramaya University, Ethiopia; e KEMRI-Wellcome Trust Research Programme, Kenya

**Keywords:** Pneumococci, Carriage, Ethiopia, Cross-sectional survey

## Abstract

**Introduction:**

Ethiopia introduced the 10-valent pneumococcal conjugate vaccine (PCV10, GlaxoSmithKline plc.) in 2011 and switched to 13-valent vaccine (PCV13, Pfizer Inc.) in 2020. In 2023, we conducted a study in four settings in Ethiopia to determine the vaccine coverage, residual vaccine-type carriage prevalence, serotype distribution, and factors associated with carriage across all age groups.

**Methods:**

A cross-sectional survey was conducted in urban and rural areas of eastern and southwest Ethiopia in 2023. In total, 50 participants in each of 10 age groups (<1, 1–2, 3–4, 5–9, 10–14, 15–19, 20–39, 40–49, 50–59, and ≥ 60 years) were randomly selected using population registers in Harar, Kersa and Gilgel Gibe demographic surveillance systems, and using random GPS points for Jimma city. After informed consent, data on socioeconomic characteristics and vaccine coverage were collected. A single nasopharyngeal swab was collected and cultured for pneumococci. Pneumococci were serotyped using latex agglutination and confirmatory Quellung reaction.

**Results:**

A total of 2006 participants were enrolled. The age-standardized population prevalence of pneumococcal carriage (all serotypes) in rural settings was 56 % (95 %CI 48–64 %) in the east and 26 % (95 %CI 20–31 %) in the southwest, and in urban settings, 15 % (95 %CI 11–20 %) in the east and 16 % (95 %CI 12–19 %) in the southwest. PCV13 serotype carriage prevalence among children aged <5 years ranged from 7.4 to 9.3 % in the urban areas, to 16–22 % in the rural areas. Coverage of the third dose of PCV, recorded in vaccination cards of participants aged <5 years, was 49 %–87 % in the urban areas; it was much lower at 13–22 % in the rural areas.

**Conclusions:**

There is considerable residual circulation of vaccine serotypes in Ethiopia, particularly in rural areas and the east, and low vaccine coverage. Pneumococcal epidemiology varies by geographical region and urban/rural setting, implying an unequal burden of pneumococcal disease across the country 12 years post-PCV introduction.

## 1 Background

Among children aged <5 years, *Streptococcus pneumoniae* (the pneumococcus) is estimated to have caused 294,000 deaths in HIV-uninfected [uncertainty range 192,000–366,000] and 23,300 deaths in HIV-infected children [UR 15300–28,700] in 2015 [1]. To reduce this high burden of pneumococcal mortality the World Health Organization recommended pneumococcal conjugate vaccine (PCV) introduction in 2007; by 2024 PCVs were included in routine immunization schedules in 166 countries in various formulations, schedules and at variable coverage [2].

Among vaccinated children, PCVs reduce susceptibility to infection in the nasopharynx (‘carriage’ or ‘colonisation’) and further reduce susceptibility to invasive pneumococcal disease (IPD) once they become colonised [3–5]. This direct protection applies only to vaccinated children and only to the serotypes included in the vaccine (VT). Current licenced vaccines contain up to 21 serotypes, but there are over 100 serotypes in nature [6,7]. PCVs also provide indirect protection from IPD for the whole population; by reducing the acquisition of VT carriage among the vaccinated, reducing the probability that all members of the population will meet with and become infected by a carrier of VT pneumococci [8,9].

PCVs reduce the prevalence of VT carriage in the nasopharynx but this facilitates colonisation by non-vaccine serotypes (NVT) [10]. In most settings, total pneumococcal carriage prevalence remains unchanged before and after vaccine introduction. This ‘serotype replacement in carriage’ can lead to NVT IPD, though, on average, NVT are less invasive than VT so the vaccine’s impact on disease incidence remains positive. However, among elderly populations in some high-income countries, serotype replacement disease has abrogated the benefits of indirect protection [10,11].

In Low- and Middle-Income Countries (LMICs), there are limited data on the serotypes responsible for IPD and even less on the impact of PCV [9,12]. In Ethiopia, modelled estimates suggest that there were approximately 15,000 pneumococcal deaths in children aged <5 years in 2015 [1]. PCV10 (Synflorix, GlaxoSmithKline plc) was introduced into the national immunization program in 2011 in a schedule of three primary doses with no booster, with a catch-up campaign for all children under-1 year of age. PCV10 was replaced by PCV13 (Prevnar13, Pfizer Inc.) in 2020 [13]. Despite over a decade of investment in expensive PCVs there has been no evaluation of the impact the program either through carriage surveys or IPD surveillance.

IPD surveillance is challenging in LMICs because it is expensive and may underestimate disease burden because of the high frequency of prior antimicrobial treatment among investigated cases [14]. Naso-pharyngeal carriage can act as an inexpensive proxy to monitor PCV impact [6,14,15] because carriage is an essential precursor for IPD [6,16] and because carriage prevalence also illustrates the indirect effects of different PCV formulations on transmission of VT and NVT [17].

We aimed to conduct cross-sectional surveys of nasopharyngeal carriage in children and adults, in representative areas of southwestern and eastern Ethiopia, in both urban and in rural populations. We aimed to determine the vaccine coverage, vaccine-type carriage prevalence, serotype distribution, and factors associated with carriage in different age groups and settings.

## 2 Methods and procedures

### 2.1 Study population

In eastern Ethiopia, we sampled residents of the Health and Demographic Surveillance Systems (HDSS) in Harar city (Harari region) and rural Kersa (Oromia region). Harar HDSS, 515 km east of Addis Ababa, had a population of 54,833 in 2023. Kersa HDSS is 44 km west of Harar with a population of 145,170 in 2023 and it contains two small towns (Kersa and Weter) [18]. In southwest Ethiopia, we selected Jimma City as an urban site and Gilgel Gibe HDSS as a rural site; both are located in Oromia region. Jimma city is 351 km southwest of Addis Ababa and had an estimated population of 263,709 in 2023 based on the last national census in 2007 [19]. Gilgel Gibe HDSS is 55 km northeast of Jimma with a population of 57,914 in 2023 [20]. The southwest exerpeiences similar average daily temperatures but has a wetter climate, than the eastern area [21].

### 2.2 Study design and sampling

A cross-sectional survey of randomly selected residents was conducted in each area between February to May 2023, which corresponds to the short rainy season (Belg). In the three HDSS sites (Harar, Kersa, and Gilgel Gibe), we applied age-stratified random sampling from the HDSS population registries. In total, 500 individuals were selected from each site, 50 from each of 10 age groups (<1, 1–2, 3–4, 5–9, 10–14, 15–19, 20–39, 40–49, 50–59, and ≥ 60 years), stratified by sex.

As there was no existing sampling frame in Jimma city, we sampled the population using randomly selected GPS coordinates. We obtained the population size of the catchment areas for the four public health centres from the Ministry of Health and marked these on high-resolution satellite images of the city from Google Earth, using ArcGIS software. 100 random points were distributed across the four polygons, weighted by the population size of each of the four areas (Supplementary Table 1, Supplementary Fig. 1). Each point’s GPS coordinates became a starting point for sampling the 5 nearest households where a resident consented to participate. When the data collectors visited the first household, they sought a person who would fit the first of the sequentially listed age strata. When they identified a household member who matched the specific age stratum, he/she was recruited. If a participant from the specific age stratum was not available in the household, the field team went to the next household to repeat this procedure. If individuals in the right age stratum refused consent, data collectors moved to the next household to replace this refusal. Once the age-stratum was sampled, the data collector moved to the next age stratum.

### 2.3 Sample size

The primary outcome of the study was the prevalence of PCV13 vaccine-type (VT) pneumococcal carriage in each of four sites. As there are no unbiased estimates of VT carriage prevalence from healthy community members in Ethiopia, we used Nigerian data to assume VT carriage prevalence of 25 % [22]. A total of 500 individuals would enable us to estimate VT carriage prevalence across each site’s population with ±4 % precision. Across the four sites, the total sample size was 2000 participants.

### 2.4 Data and sample collection

After obtaining written informed consent we collected socio-demographic data and risk factors for pneumococcal acquisition on tablets using REDCap electronic data capture tools [23,24] in the local language. Nasopharyngeal samples were collected with sterile, flexible, nylon-tipped flocked swabs (Copan Flock Technologies, Cat. No.503CS01, Italy) in accordance with the WHO guideline [25]. Swabs were placed immediately in 1 mL skimmed-milk tryptone glucose glycerol (STGG) transport media and transported in a cool box at 2–8 °C to Hararghe Health Research laboratory (HHRL), Haramaya University, for the Eastern sites, and to Jimma University Laboratory, for the Southwest site, where they were stored at –80 °C within 8 h of collection. Samples stored at Jimma University were shipped at –80 °C to Haramaya University at the end of the survey.

### 2.5 Laboratory methods

At HHRL, STGG samples were inoculated onto 5 % horse blood agar with gentamicin and incubated at 37 °C in 5 % CO_2_ overnight. Pneumococci were identified by alpha haemolysis, optochin sensitivity, and bile solubility testing. Isolates were stored in Trypton-soy broth with 15 % glycerol at −70 °C [26] for further analysis.

Nasopharyngeal samples were shipped on dry ice to the KEMRI-Wellcome Trust Research Laboratory (KWTRL) in Kenya for re-culture and serotyping. The culture results were compared across the labs to validate the procedures at HHRL. At KWTRL, one colony of pneumococci on each plate was selected at random for serotyping by latex agglutination and confirmatory Quellung reaction. Polymerase Chain Reaction (PCR) was performed for quality control purposes on a 10 % random sample of the cultures at KWTRL, as per institutional quality assurance plans, and as a confirmatory test for samples that had ambiguous Quellung tests. Detection of lytA by PCR was used to determine the potential presence of pneumococci and multiplex PCR was performed to serotype isolates during quality control.

### 2.6 Statistical analysis

For all children aged <5 years, data on vaccine coverage was gathered from their vaccine booklet (card).Vaccine coverage for PCV was calculated as follows: -

$$\text{Vaccine}\;\text{coverage}\;\text{by}\;\text{booklet}=\frac{number\;of\;children\;who\;had\;the\;vaccine\;recorded\;in\;their\;books}{number\;of\;children\;with\;vaccination\;books}$$

Overall carriage prevalence (of any pneumococci), VT, and NVT carriage prevalence, was calculated across all ages and in age strata. VTs were classified as serotypes in PCV13 (1, 4, 5, 6B, 7F, 9 V, 14, 18C, 19F, 23F, 3, 6 A, 19 A). When combining carriage prevalence from different age strata to form larger age-groups, the carriage prevalences were weighted using the population age structures in 2023 obtained from the HDSSs; as there was no HDSS in Jimma, the Harar city HDSS population age structure was applied to the results from Jimma city. Simpsons Index of Biodiversity (D) was calculated as follows to measure the diversity of serotypes and was compared to samples of pneumococci from other countries: D = 1 – (∑ *n*(*n*-1)) / N (*N*-1)), where *n* is the number of individuals displaying one trait (species or serotype) and N = the total number of all individuals.

Sociodemographic characteristics were assessed for their association with carriage (all pneumococci) using logistic regression, controlling for three variables (age, sex, and site) as a priori confounders due to the sampling strategy. Factors that were associated with carriage in crude analyses (*p*-value ≤0.05) were then considered for inclusion in the multivariable logistic regression model. Stepwise selection procedures were applied to the multivariable regression model. Initially, all variables with a p-value ≤0.05 in the univariable analysis were added to the model, and then variables were sequentially removed to test their association in the final model. Excluded variables were added back in to the final model to check for residual confounding. Only variables with a likelihood ratio test (LRT) p-value of ≤ 0.05 remained in the final model.

The research ethics committees of Haramaya University, Jimma University, the Ethiopian National Ethical Review Committee and the London School of Hygiene & Tropical Medicine approved the study. All participants gave written informed consent except children aged <18 years where written consent was obtained from their parents/guardians. Participants aged 12–17 years old also provided written informed assent.

## 3 Results

### 3.1 Participant characteristics

A total of 2311 households were visited, 245 household members were either absent, had out-migrated or died. Among 2066 household members approached to participate, consent was obtained from 2006 (97 %, Supplementary Table 2).

An equal number of males and females were recruited in the HDSS sites, where it was possible to stratify the sample by sex; however, in Jimma, males were found at home less frequently and were only 34 % of the participant population (Table 1). In rural areas, 65–91 % of the household heads were farmers, whereas in urban areas, 64–70 % of household heads were merchants or professionals. Rural participants lived in larger households; 40–53 % lived with >4 other people, compared to 16–29 % in urban areas. In rural areas, only 2–15 % of households ever used electricity/gas for cooking, whereas in urban areas, 39–59 % of households used electricity/gas; all households across all sites used some solid fuels i.e. wood/charcoal for cooking, if needed (Table 1, Supplementary Tables 3 & 4).

### 3.2 Vaccine coverage

Vaccination booklet retention among children <5 years of age was high across all sites; it was lowest in Jimma City at 70 %. Among children aged <5 years with vaccination booklets, coverage of the third dose of PCV (scheduled at 14 weeks of age) was 13 % in Gilgel Gibe, 49 % in Jimma, 22 % in Kersa and 87 % in Harar (Table 2). Third-dose PCV coverage among children <1 year of age followed a similar pattern across the sites, it was only 9 % in Gilgel Gibe, 50 % in Jimma, 18 % in Kersa and 87 % in Harar (Supplementary Table 5). There is no adult PCV programme in Ethiopia.

### 3.3 Pneumococcal carriage prevalence

**Fig. 1:**
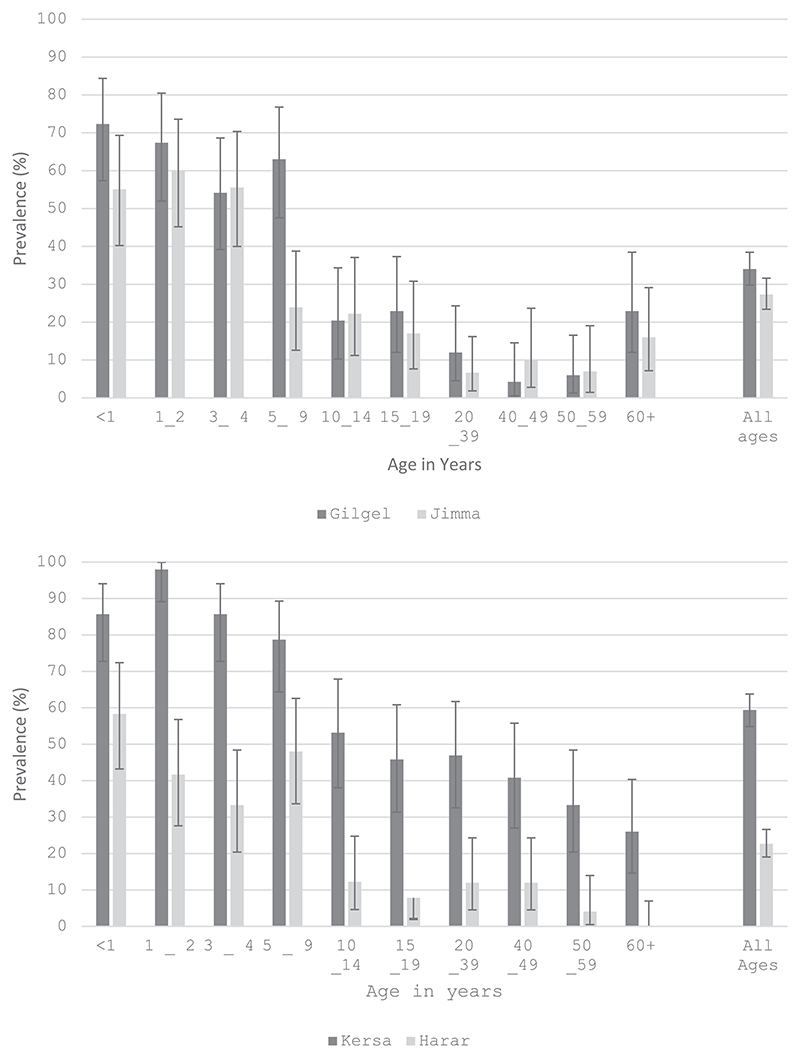
Observed prevalence of pneumococcal carriage (all serotypes) by Age and Location.^1^. ^1^Pale bars are urban sites, dark bars are rural sites.

Among those who consented, swabs were collected from 2003 participants and successfully cultured from 1989 participants (99 %), 745 (37.5 %) yielded pneumococci and were serotyped. The results with serotype-specific data from KWTRL, were used in the analysis below. The crude pneumococcal carriage prevalence (all serotypes) was 34 % (163/479) in Gilgel Gibe, 27 % (130/475) in Jimma, 59 % (288/485) in Kersa and 23 % (112/494) in Harar. After adjusting for the age-stratified sampling schema using the local population structures as weights, age-standardized carriage prevalence was 25.6 % (95 %CI 20.3–30.9) in Gilgel Gibe, 15.6 % (95 %CI 11.8–19.3) in Jimma, 55.6 % (95 %CI 47.7–63.5) in Kersa, and 15.2 % (95 %CI 10.9–19.5) in Harar (Supplementary Table 6). Pneumococcal carriage prevalence (all serotypes) was notably higher in infants and children aged 1–2 years compared to older age groups across the four locations (Fig. 1).

In the validation of pneumococcal culture techniques at HHRL, using KWTRL as the reference standard, culture sensitivity was 91 %, and specificity was 96 %, (Supplementary Table 7).

### 3.4 Vaccine-type carriage prevalence

Residual PCV13-type carriage prevalence remained high in children aged <5 years in rural areas; the age-standardized prevalence was 16.3 % (95 %CI 15.7–16.9) in Gilgil Gibe, 22.4 % (95 %CI 21.3–23.5) in Kersa. However, PCV13-type prevalence was lower in the urban areas; age-standardized prevalence was 7.4 % (95 %CI 7.05–7.74) in Jimma, 9.2 % (95 %CI 8.86–9.57) in Harar. PCV13-type carriage prevalence decreased with age across all sites (Table 3 and supplementary Tables 6 & 8). Only a small proportion of this PCV13-type carriage was due to the additional serotypes in PCV13; serotypes 3, 6 A, 19 A were < 1 % prevalence in rural areas and 2.1–2.5 % prevalence in urban areas. The age-standardized prevalence of carriage with the seven additional serotypes contained in PCV20 was 7–12 % in rural sites and 8–12 % in urban sites.

In adults (aged 15 years or above), vaccine-type carriage prevalence was low; in urban sites and the south-west rural site 3.8–4.4 % carried PCV20 serotypes; in the eastern rural site 9.9 % carried PCV20 serotypes (Table 3).

### 3.5 Serotype-specific carriage prevalence

Among the 695 pneumococcal isolates from all sites, 67 serotypes were identified. In the urban populations, 48 different serotypes were identified among 244 pneumococcal isolates, and in the rural population, 65 different serotypes were identified among 451 pneumococcal isolates. Simpson’s Index of Diversity (D) was 0.96 in the urban areas and 0.98 in the rural areas, emphasizing wide diversity within the species in both settings (Supplementary Table 9).

**Fig. 2:**
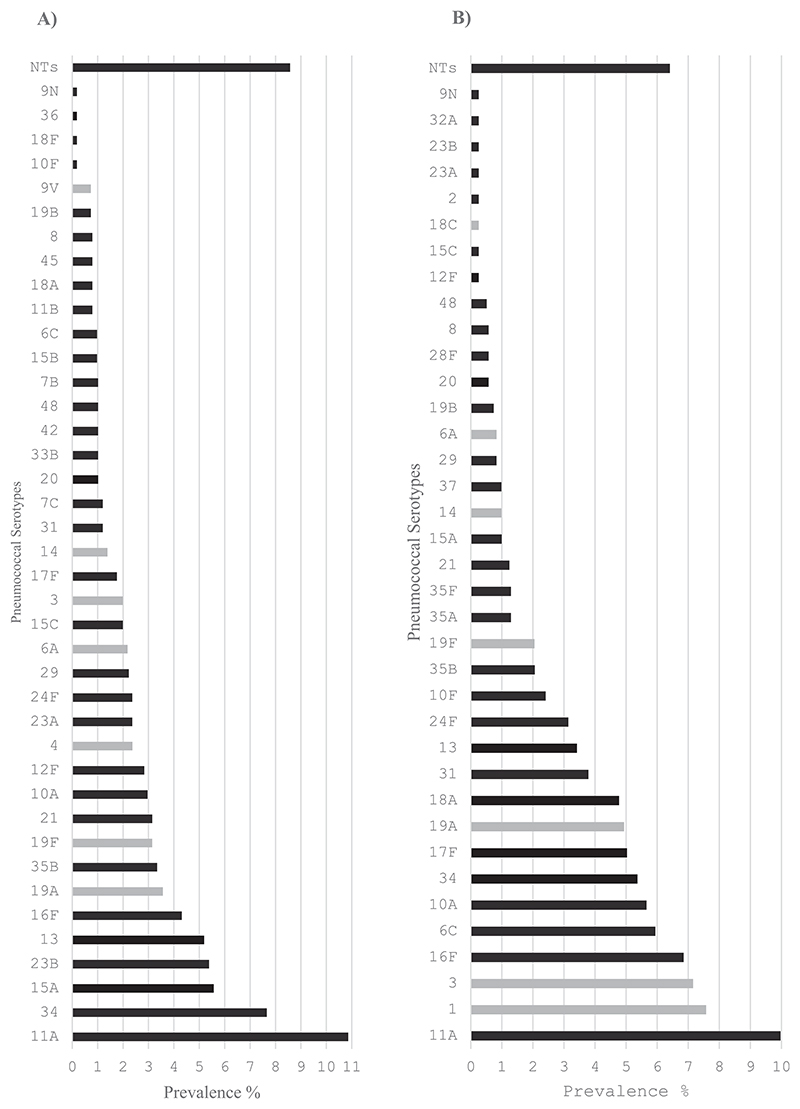
Age-standardized serotype-specific prevalence among Ethiopian Urban populations in 2023 in A) Children aged <5 years and B) other participants aged 5 years or older^1^. ^1^Light grey bars denote PCV13 VTs, dark grey bars denote non-VTs. NTs = non-typeables.

**Fig. 3:**
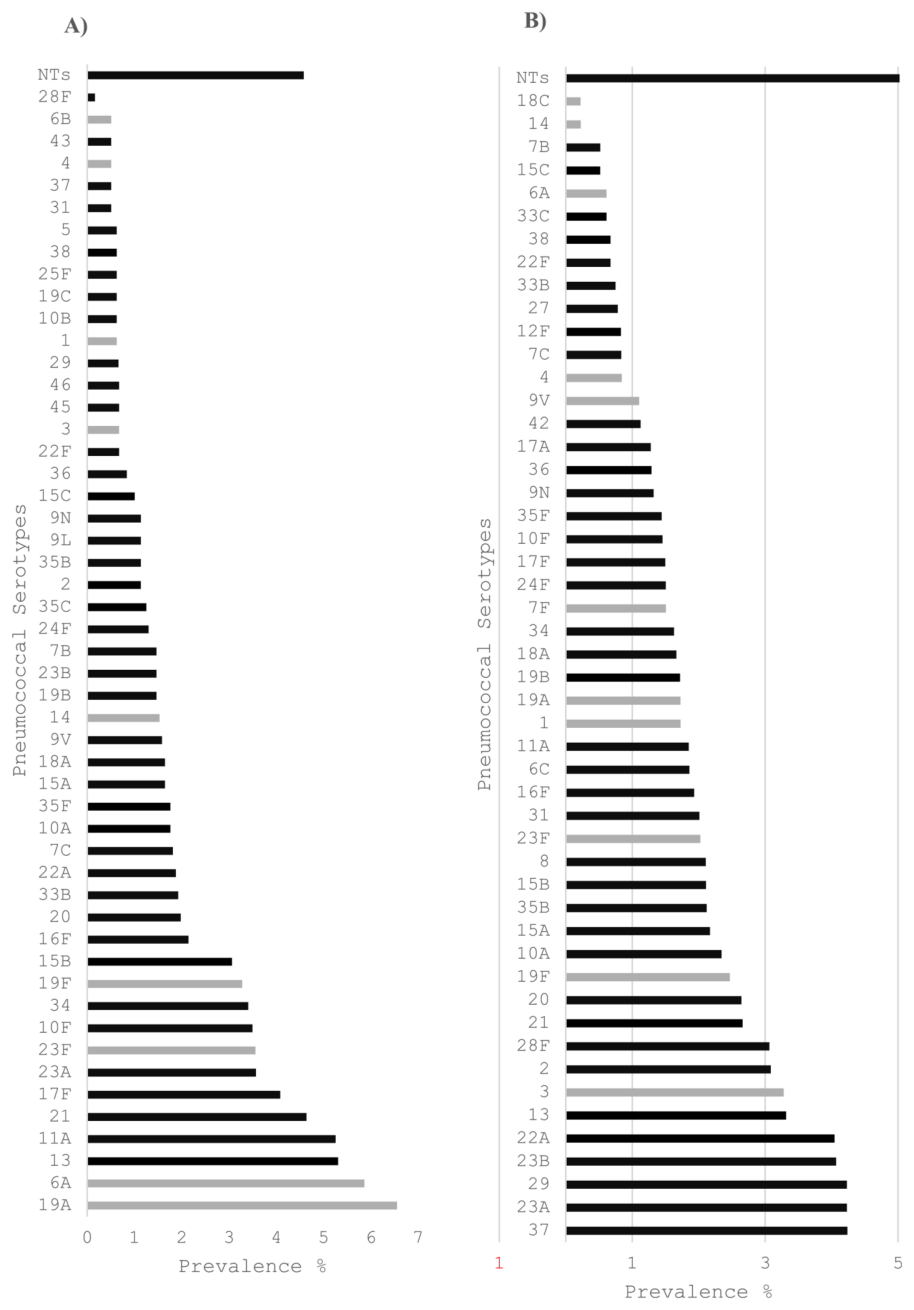
Age-standardized serotype specific prevalence of *S. pneumoniae* among Ethiopian Rural populations in 2023 in A) children aged <5 years of age and B) all other participants aged 5 years or older^1^. ^1^Light grey bars denote PCV13 VTs, dark grey bars denote non-VTs. NTs = non-typeables.

Among children aged <5 years living in urban areas, the ten most common serotypes in rank order were: 11 A (10.9 %), 34 (7.7 %), 15 A (5.6 %), 23B (5.4 %), 13 (5.2 %), 16F (4.3 %), 19 A (3.6 %), 35B (3.3 %), 19F (3.1 %), and 21 (3.1 %). In rural areas, the ten most common serotypes were: 19 A (6.6 %), 6 A (5.9 %), 13 (5.3 %), 11 A (5.3 %), 21 (4.6 %), 17F (4.1 %), 23 A (3.6 %), 23F (3.6 %), 10F (3.5 %), and 34 (3.4 %) (Figs. 2, 3, Supplementary Table 10).

Among persons aged ≥5 years living in urban areas, the ten most common serotypes were 11 A (10.0 %), 1 (7.6 %), 3 (7.2 %), 16F (6.9 %), 6C (5.9 %), 10 A (5.7 %), 34 (5.4 %), 17F (5.0 %),19 A (4.9 %), 18 A (4.8 %), and 31 (3.8 %). In rural areas the ten most common serotypes were 37 (4.2 %), 23 A (4.2 %), 29 (4.2 %), 23B (4.1 %), 22 A (4.0 %), 13 (3.3 %), 3 (3.3 %), 2 (3.1 %), 28F (3.1 %), and 21 (2.7 %) (Figs. 2, 3 & supplementary Table 11).

### 3.6 Factors associated with carriage of any serotype

In crude analyses, age, sex, and area (Gilgel Gibe, Jimma, Kersa, and Harar) were included as a priori variables. In crude analyses, educational status of the head of the house, number of windows and people in the household, respiratory tract symptoms and contact with preschool children were all associated with pneumococcal carriage of any serotype (Supplementary Table 12). In multivariable analyses, significant independent predictors of carriage of any serotype included area, age, educational status of the head of the household, number of windows in the household and contact with preschool children (Supplementary Table 12). The covariates, sex, mother’s education, number of rooms and people living in the household, cooking place, frequency of cigarette smoking, bed net usage, respiratory tract symptoms and contacts with infants were not associated with carriage when all other factors were controlled for.

## 4 Discussion

This is the first population-based survey conducted in Ethiopia among children and adults to estimate serotype-specific carriage prevalence. The results indicate very diverse population prevalences of all-serotype carriage across the areas sampled, i.e. a much higher prevalence in rural areas and the southwest, compared to the eastern city of Harar, after accounting for all other relevant factors. All-serotype carriage prevalences of >50 % extended from infancy up to the age of 10 or 14 years in rural areas whereas, in urban areas, carriage prevalences >50 % were restricted to children aged <5 years. We found substantial diversity of serotypes in both urban and rural settings (D = 0.96–0.98). These results are consistent with studies from Northern and Central Ethiopia (D = 0.98) [27], but represent subtaintially higher diversity than reported in similar cross-sectional surveys in rural Kenya (D = 0.93) [28], urban Nigeria (D = 0.91) [22]and rural Nigeria (0.86) [22].

Twelve years after the introduction of PCV10 (GSK) and three years after the switch from routine immunization with PCV10 to PCV13, residual PCV13 type carriage remains high in children aged <5 years in rural areas (16–22 %); however, it was lower in the urban areas (7–9 %). The extremely low coverage of PCV, except in Harar city, is likely to be contributing to both the high diversity of circulating serotypes, and the high levels of residual VT carriage. The very low PCV coverage we observed (9–50 % in infants) is consistent with the 2019 Ethiopian Demographic Health Survey which documented 20–50 % third-dose PCV coverage in both Jimma and Harari regions, and also indicated coverage is significantly lower in rural areas than urban areas (nationally, third-dose PCV coverage was 67 % in urban areas, and 38 % in rural areas) [29].

The variability in PCV coverage doesn’t explain all the variability in VT carriage prevalence among children <5 years of age across the distinct areas sampled. In both the eastern and southwestern areas, we documented higher VT carriage and lower vaccine coverage in the rural area, compared to the urban area. However, VT carriage was higher in both eastern sites than the in the comparator sites in the southwest, despite higher vaccine coverage. Distinct setting-specific relationships between VT-carriage and PCV coverage have also been documented elsewhere [9,30]. Other factors, which influence the force of infection with VT, are likely to influence VT carriage prevalence i.e. host susceptibility, the quality of vaccine-induced immunity, local serotype distribution of circulating pneumococci, and migration [31]. In our multivariable regression analysis we found the educational status of the household head and the number of windows in the household, perhaps indicators of wealth and/or sanitation, decreased the odds of carrying any pneumococci, after controlling for all other factors including site. Higher numbers of contacts with pre-school children, known to be transmitters of pneumococci, increased the odds of carrying any pneumococci, after controlling for all other factors. Other studies in Ethiopia have also indicated that household proxies for wealth and crowding, were independent predictors of carriage [31–34].

Despite the very low PCV coverage recorded in this study, VT carriage prevalence appears to be slightly lower now, across all of the sites, compared to a study in northern Ethiopia in 2007, pre-vaccine introduction, among children 0–9 years of age enrolled in a control group of a cluster randomised trial [35]. In 2007 in northern Ethiopia, PCV13-type carriage prevalence was 36.8 % (95 % CI, 28.0 %–45.4 %), whereas in 2023 we found the age-standardized prevalence ranged from 9.5 to 30 % among 0–9-year-olds in eastern and southwestern Ethiopia. Given the differences in study design we can’t attribute this to vaccine introduction alone.

This is the first study conducted in Ethiopia to investigate the serotype distribution among adult populations. In this population (≥15 years of age), carriage of any pneumococci was relatively common (age standardized prevalences of 13–43 % in rural areas, 9–10 % in urban areas), but only a small proportion of carriage was with serotypes included in some of the new high valency vaccines e.g. PCV20. The prevalence of PCV20 types was 4–10 % in rural areas and 4 % in urban areas. Among NVTs, distinct serotypes were identified across sites and age groups. Data on serotype replacement disease from LMICs are scarce [12]. Studies that identify serotype-specific carriage prevalence could be used alongside serotype-specific invasiveness estimates to estimate serotype-specific invasive pneumococcal disease incidence, given that carriage is a prerequisite for the disease [6,9]. This approach may be useful to predict the potential impact of switching product or schedule on circulating VT and NVT within the Ethiopian population.

Our study was limited to two geographical areas, four distinct populations, of Ethiopia and does not represent the diverse enrironments across the country. Almost all swabs were frozen soon after collection, and cultured and serotyped at a later date; which could have compromised the results of culture and serotyping; however, this will not have confounded the comparisons across sites in this analysis. Only the first 100 swabs from Harar city were cultured on arrival at HHRL, prior to freezing, for QC purposes. No recall data were collected from participants who were missing vaccination cards, this would have been especially useful in Jimma city where 30 % of respondents were missing vaccination cards. Those missing cards may be more likely to have missed vaccination sessions and therefore we may have overestimated vaccine coverage in Jimma city (third-dose PCV coverage could be 33 % rather than 47 % if all those missing cards were actually unvaccinated).

## 5 Conclusion

This study illustrates considerable residual circulation of serotypes currently targeted by the PCV13 national immunization program, particularly in rural areas and the eastern sites, which could be due to very low levels of vaccine coverage. It also demonstrates wide diversity in pneumococcal epidemiology in different geographical regions and rural and urban settings. Socioeconomic factors like residence area, age, household heads’ educational status, number of windows in the household, and contact with preschool children, influence carriage prevalence of any serotype. The large sample size, inclusive of all ages, and the representative random sampling make these results suitable for modelling vaccine impact to guide future vaccine policy in Ethiopia.

## Figures and Tables

**Table 1:** Participant Characteristics among participants with swabs taken (*n* = 2003).

Characteristics	Site													
	Southwest						Eastern							
	Gilgel Gibe (Rural)			Jimma (Urban)			Kersa (Rural)			Harar (Urban)			Total	
	N	%		N	%		N	%		N	%		N	%
**Sex**														
Male	231	46		168	34		245	49		242	48		886	44
Female	266	54		332	66		257	51		261	52		1116	56
**Occupation of the head of the household**														
Farmer	320	65		33	7		457	91		21	4		831	42
Merchant	70	14		238	50		7	2		150	30		465	24
Professional	47	10		67	14		3	1		199	40		316	16
Other/Child	56	11		142	30		27	5		128	26		353	18
**The main source of income for the household**														
Employed	39	8		95	19		8	2		194	39		336	17
Self-Employed	440	89		353	71		407	92		221	44		1421	73
Other	16	3		51	10		28	6		84	17		179	9
**Number of other people living in the household (excluding the participant)**														
0	46	9		35	7		38	8		49	10		168	8
1−4	252	51		386	77		194	39		307	61		1139	57
5−8	174	35		74	15		218	43		136	27		602	30
9−13	26	5		5	1		52	10		11	2		94	5
**Energy source for cooking^1^**														
Kerosine	0	0		36	7		1	0		48	10		85	4
Electricity/Gas	76	15		196	39		8	2		294	59		574	29
Wood/charcoal	482	97		436	87		477	95		447	89		1842	92
**Frequency of cigarette smoking in the household**														
Daily/weekly	37	7		7	1		268	55		66	13		378	19
Never/Rarely	459	93		493	99		224	46		436	87		1612	81
Total Sample size^2^	498			500			502			503			2003	

1 Participants could provide multiple answers for energy source for cooking.

2 Sample size varies for each variable due to missing data.

**Table 2:** Coverage of PCV among study participants aged <5 years by dose and site.

									Vaccine booklet alone^a^			
Site		Dose		Sampled		Missing vaccine booklet^a^			Received		Not received	
				**N**		**n**	**%**		**n**	**%**	**n**	**%**
Southwest												
Gigel Gibe		PCV1		150		8	5.3		24	16.9	118	83.1
(rural)		PCV2		150		8	5.3		23	16.2	119	83.8
		PCV3		150		8	5.3		19	13.4	123	86.6
Jimma		PCV1		153		43	28.1		56	50.9	54	49.1
(urban)		PCV2		153		43	28.1		53	48.2	57	51.8
		PCV3		153		46	30.1		50	46.7	57	53.3
East												
Kersa		PCV1		151		5	3.3		42	28.8	104	71.2
(rural)		PCV2		151		6	4.0		36	24.8	109	75.2
		PCV3		151		5	3.3		32	21.9	114	78.1
Harar		PCV1		150		3	2.0		131	89.1	16	10.9
(urban)		PCV2		150		4	2.7		126	86.3	20	13.7
		PCV3		150		3	2.0		128	87.1	19	12.9

a Those missing a vaccination booklet were excluded from denominator when calculating vaccine coverage based on vaccine booklet data alone. There were no additional recall data available from those who were missing a booklet (all recall data were missing for participants aged <5 years who were missing a vaccine booklet), so complete vaccine coverage was not calculated.

**Table 3:** Age-standardized prevalence of all serotypes and specific serogroups by age group.

	N	Crude Carriage Prevalence (all serotypes)	Age-standardized carriage prevalence (all serotypes)	Age-standardized carriage prevalence (PCV13 VT types)	Age standardized carriage prevalence (PCV 15 VT types)	Age standardized carriage prevalence (PCV 20 VT types)	Age standardized carriage prevalence (added 3 serotypes in PCV13)^1^
**Gilgil Gibe HDSS, South-west Rural site**							
**<5** **years**	141	64.5	60.2	16.3	16.7	23.8	0.8
**5−14 years**	95	41.1	43.2	4.4	5.6	10.2	1.2
**≥** **15** **years**	243	13.6	13.4	3.3	3.7	3.8	0.9
**Jimma City, South-west Urban site**							
**<5** **years**	144	56.9	57.4	7.4	7.4	19.8	2.5
**5−14 years**	91	23.1	23	4.4	4.4	9.8	2.2
**≥** **15 years**	240	11.3	9.7	3.7	3.7	4.4	0.9
**Kersa HDSS, Eastern Rural site, Kersa HDSS**							
**<5** **years**	147	89.8	91.3	22.4	23.3	34.8	0.9
**5−14 years**	94	66.0	66.3	12.8	12.8	23.6	3.2
**≥** **15 years**	244	38.5	42.9	6.3	6.8	9.9	1.8
**Harar HDSS, Eastern Urban site**							
**<5** **years**	144	44.4	39.6	9.2	9.2	17.3	2.1
**5−14 years**	99	30.3	29.6	8.9	8.9	10.9	4.9
**≥** **15 years**	251	7.2	9.3	1.5	2.0	4.4	0.9

1 Serotypes 3, 6 A, 19 A.

## Data Availability

The data will be held in the LSHTM data repository. Data access will be granted upon reasonable request. Reasonable request is defined as: The requestor has a disclosed hypothesis and research question that can be answered using the data and is affiliated with a reputable research organisation, which has capacity to store and analyse the data according to good clinical practice/good data management practice. All potential users wishing to obtain the data must complete a request form: (http://datacompass.lshtm.ac.uk/cgi/request_doc?docid=1).

## Footnotes

**Appendix A. Supplementary data**
Supplementary data to this article can be found online at https://doi.org/10.1016/j.vaccine.2025.127762.
